# List of Diseases from the 20th of March to the 20th of April; Being the Result of the Public and Private Practice of a Physician at the West End of the Town

**Published:** 1799-05

**Authors:** 


*List of Diseases from the 20th of March to the 20th of April; being the Result of the public and private Practice of a Physician at the West End of the Town.*

| ACUTE DISEASES. | No. of Cases. | | No. of Cases. |
|---|---|---|---|
| Catarrh - - - | 22 | Hysteria - - - - | 3 |
| Acute Rheumatism - - | 5 | Epilepsy - - - - | 2 |
| Pleuritic Stitches - - | 3 | Chorea - - - - | 2 |
| Peritoneal Inflammation - | 1 | Paralysis - - - | 6 |
| Inflammatory Sorethroat | 4 | Cephalæa - - - | 8 |
| Specky Sorethroat - | 2 | Epistaxis - - - | 1 |
| Ophthalmia - - - | 7 | Dyspepsia - - - | 10 |
| Scarlatina - - - | 6 | Gastrodynia - - - | 5 |
| Measles - - - | 3 | Enterodynia - - | 4 |
| Small-pox - - - | 3 | Diarrhœa - - - | 8 |
| Malignant Fever - - | 5 | Colica Pictonum - - | 2 |
| Slow Fever - - - | 6 | Hæmorrhoids - - | 3 |
| Hectica - - - | 7 | Jaundice - - - | 2 |
| Child-bed, and Milk-fever | 2 | Fluor albus - - - | 4 |
| Acute Diseases of Infants | 8 | Chlorosis - - - | 8 |
| Tertian - - - | 1 | Menorrhagia - - | 3 |
| | | Abortion - - - | 1 |
| | | Worms - - - | 3 |
| CHRONIC DISEASES. | | Tabes Mesenterica - | 3 |
| Cough and Dyspnoea - | 79 | Scirrhus - - - | 2 |
| Hæmoptoe and Phthisis - | 18 | Itch and Prurigo - - | 74 |
| Chronic Rheumatism - | 12 | Lepra - - - - | 1 |
| Lumbago and Sciatica - | 5 | Nettle-rash - - - | 1 |
| Dropsy - - - | 9 | Purpura - - - | 2 |
| Scrophula - - - | 5 | Gutta Rosea - - - | 3 |
| Asthenia - - - | 21 | Porrigo - - - | 2 |

The measles are at present declining: in one child, who had been previously affected with the hooping-cough, the rash was succeeded by numerous livid spots, diffused over almost the whole body, and resembling those of the purpura, or the petechiæ sine febre, in their most dangerous form. No harm, however, ensued; and the complaint was removed in about eight days.

The scarlatina has, during this present month, become much milder, so that all the cases put down, terminated early and favourably.

Inflammation of the eyes, next to catarrh, cough, and rheumatism, seems to have been the most general disease; it was very troublesome, and in many cases, of long duration. The same complaint occurred epidemically in the months of February, March, and April, of the last year.

It may not be amiss to observe, that the itch, which is usually rife in the spring-season, has, during the last six weeks, taken a very wide range, and appeared in many respectable families, never before liable to the intrusion of such a visitor. R. W.

Account

